# Can a plant biologist fix a thermostat?

**DOI:** 10.1111/nph.20382

**Published:** 2025-01-02

**Authors:** Todd P. Michael

**Affiliations:** ^1^ Plant Molecular and Cellular Biology Laboratory The Salk Institute for Biological Studies La Jolla CA 92037‐100210 USA

**Keywords:** artificial intelligence (AI), CRISPR‐CAS9, large language models (LLMs), next‐generation sequencing (NGS), pangenome, plant artificial chromosomes (PACs), single‐cell sequencing (scRNA‐seq), telomere‐to‐telomere (T2T)

## Abstract

The shift to reductionist biology at the dawn of the genome era yielded a ‘parts list’ of plant genes and a nascent understanding of complex biological processes. Today, with the genomics era in full swing, advances in high‐definition genomics enabled precise temporal and spatial analyses of biological systems down to the single‐cell level. These insights, coupled with artificial intelligence‐driven *in silico* design, are propelling the development of the first synthetic plants. By integrating reductionist and systems approaches, researchers are not only reimagining plants as sources of food, fiber, and fuel but also as ‘environmental thermostats’ capable of mitigating the impacts of a changing climate.

**Table:** 

	Contents	
	Summary	1403
I.	Introduction	1403
II.	References to pangenomes	1404
III.	The rise of high‐definition genomics	1404
IV.	The *in silico* plant emerges	1406
V.	Designing the synthetic plant	1407
VI.	Can a plant biologist fix a thermostat?	1408
	Acknowledgements	1408
	References	1408

## I. Introduction

In the thought‐provoking commentary ‘Can a biologist fix a radio’? (Lazebnik, 2002), Yuri Lazebnik explores the limitations of applying reductionist techniques, such as gene knockouts, to understand and troubleshoot complex systems. He imagines a scenario where a molecular biologist, using standard reductionist methods, attempts to understand how a radio works. In this analogy, removing (or ‘knocking out’) a transistor causes the radio to stop working, leading the biologist to conclude that the transistor is the most crucial component. While simplistic, this analogy highlights the limitations of reductionism for understanding complex systems. Lazebnik ultimately questions whether reductionist techniques alone are sufficient for truly understanding complex biological systems (Lazebnik, 2002).

In a follow‐up study, Jonas & Kording (2017) build on Lazebnik's thought experiment by applying experimental approaches commonly used in neurobiology to see if they could understand and ‘fix’ a microprocessor. Using a variety of reductionist techniques typically employed in neuroscience, they attempted to analyze a well‐understood microprocessor. Their findings echo Lazebnik's conclusion that these reductionist methods fall short in providing a comprehensive understanding, even of a system far simpler than the brain.

Plant biologist Sophien Kamoun brought this analogy full circle in his commentary, ‘Can a Biologist Fix a Smartphone?—Just Hack It!’ (Kamoun, 2017). Kamoun reframes the analogy to include both the ‘software’ (DNA) and the ‘hardware’ (the cell), while adding an evolutionary perspective. He suggests that the true potential for fixing a complex system lies in manipulating the ‘software’ using gene‐editing tools we now possess, allowing us to effectively ‘hack’ biological systems. This synthesis brilliantly captures the current frontier in plant research and underscores the synergy between reductionist and systems approaches for understanding complex traits (Williams & Auwerx, 2015).

How can we truly understand complex systems? Jonas & Kording (2017) argue that it is not simply a matter of collecting more data, but instead creating specific, functionally informative data that uncovers the intricate interconnections within these systems. Advances in next‐generation sequencing (NGS) are delivering ‘high‐definition genomics’, including telomere‐to‐telomere (T2T) pangenomes with single‐cell functional annotations. These comprehensive datasets are now powering emerging artificial intelligence (AI) tools, such as large language models (LLMs), which are poised to transform our understanding of plant biology. Together, these innovations enable the modeling of plants *in silico* and lay the groundwork for designing synthetic plants using genome‐editing technologies such as CRISPR‐Cas9 and pioneering developments such as plant artificial chromosomes (PACs). These breakthroughs have the potential to revolutionize agriculture by improving how we feed, clothe, and fuel the world while addressing the urgent need to regulate our planet's ‘thermostat’ in the face of climate change.

## II. References to pangenomes

One of the broad shifts in plant biology was a movement away from multispecies ‘spray and pray’ experiments toward reductionist approaches in model species to understand how plants work at the mechanistic level. While the duckweed family played an important role in the beginning of model‐based plant research (Hillman, 1976), they were superseded by what we now know as the model plant species *Arabidopsis thaliana* (Meyerowitz, 1989). As was seen by Lazebnik in his field of apoptosis, the introduction of Arabidopsis as a model species led to ‘gold rush’ of new gene discoveries and understanding of plant molecular processes (Rhee & Mutwil, 2014), which were supercharged by the sequencing and publication of the genome in 2000 (Arabidopsis Genome Initiative, 2000). Over the past 20 yr, Arabidopsis has become the foundation for much (not all) plant research with scientists on wild and crop plants citing Arabidopsis gene orthologs as a way of understanding their findings (International Arabidopsis Informatics Consortium, 2019). However, now that we are rapidly approaching 1000 plant genomes (Sun *et al*., 2022), we can see that the whole genome duplication (WGD) history and retention of multigene families in Arabidopsis means that its genome may not always translate to other species of interest in contrast to species in other clades with more representative genomes (Lam & Michael, 2022).

**Fig. 1:**
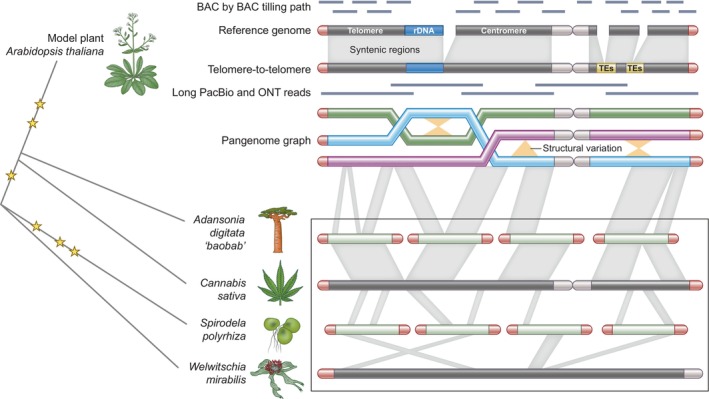
From reference genomes to pangenomes of plants with novel architectural, biochemical and evolutionary features. Most plant reference genomes were Sanger sequenced BAC by BAC, which resulted in high‐quality genomes that were missing some of the most difficult repetitive sequences to assemble such as the telomere (red), centromere (gray), ribosomal DNA (rDNA, blue) and transposable elements (TEs, yellow). Reference genomes were later improved with single‐molecule long‐read sequencing technologies such as Pacific Bioscience (PacBio) and Oxford Nanopore Technologies (ONT) that enabled the completion of the hard to assemble parts of the genome and ultimately the telomere‐to‐telomere (T2T) assemblies. At the same time, the cost of sequencing decreased substantially leading to the opportunity to sequence many plants of the same species or genus, paving the way for pangenomes and superpangenomes that capture broad structural variation (SV, orange) of a species. The combination of long‐read sequencing, declining costs, and advanced *ab initio* gene prediction tools has revolutionized plant genomics, enabling the sequencing of a diverse array of plants beyond traditional models and crops. These efforts span the evolutionary gradient of the green kingdom, capturing species with unique biology (e.g. *Adansonia digitata*, the long‐lived baobab tree), untapped biosynthetic potential (*Cannabis sativa*, known for its medicinal and industrial applications), distinctive lifestyles and architecture (*Spirodela polyrhiza*, a member of the duckweed family representing early non‐grass monocots with the smallest and fastest‐growing plants), and remarkable photosynthetic adaptations (*Welwitschia mirabilis*, adapted for extreme water‐use efficiency and photosynthesis in arid desert environments). High‐quality genomes enable the identification of whole genome duplication (WGD) events over evolutionary time (yellow stars), which have profoundly influenced genome architecture, gene content, and population structure. These WGD events provide a template for understanding how natural selection has shaped plant genomes to exploit specific environmental niches (box: syntenic blocks in gray across evolutionary time). This evolutionary ‘road map’ offers valuable insights into solutions to many of the challenges we face today, serving as a foundation for developing the first generation of synthetic plants.

Advances in sequencing revolutionized the reference‐based paradigm of plant research (Fig. 1). The advent of short‐read sequencing initiated a gradual influx of highly fragmented genomes from less‐studied species, reintroducing opportunities to explore the evolutionary dynamics of complex biological systems (Michael & Jackson, 2013). However, many of these studies still relied heavily on Arabidopsis as a reference due to the growing knowledge and tools developed in that system such as knockout lines that enabled rapid testing of gene hypotheses (Alonso *et al*., 2003). The general genome assembly quality increased with the introduction of single molecule long‐read sequencing (Michael & VanBuren, 2020), resulting in the first near‐complete genome (VanBuren *et al*., 2015) and higher quality versions of Arabidopsis for a fraction of the cost in days not years (Michael *et al*., 2018). Ultimately, these technologies led to a T2T version of Arabidopsis (Naish *et al*., 2021) and large studies to understand the evolution, relationships, and conservation of plant genes (Zhao & Schranz, 2019).

Pangenomes and super‐pangenome are emerging as the new reference (Bayer *et al*., 2020). While resequencing with short reads provided a broad view of the variation in a species such as the Arabidopsis 1001 project (1001 Genomes Consortium, 2016), it is becoming clear that the level of structural variation (SV) missed by focusing on one reference genome impacts breeding and improvement efforts (Lian *et al*., 2024). Pangenomes, whether reference‐based or reference‐free, are increasingly constructed using advanced computational approaches such as graph‐based methods based on hierarchical multi‐sequence alignment (Armstrong *et al*., 2020; Garrison *et al*., 2023) or scalable k‐mer‐based methods, which can handle thousands of genomes (Aylward *et al*., 2023). The convergence of technologies enabling the generation of numerous high‐quality genomes for a species or genus, combined with emerging reference‐free (super)pangenome computational methods, reinforce the need for many genomes to represent the genome architecture of a species.

## III. The rise of high‐definition genomics

**Fig. 2:**
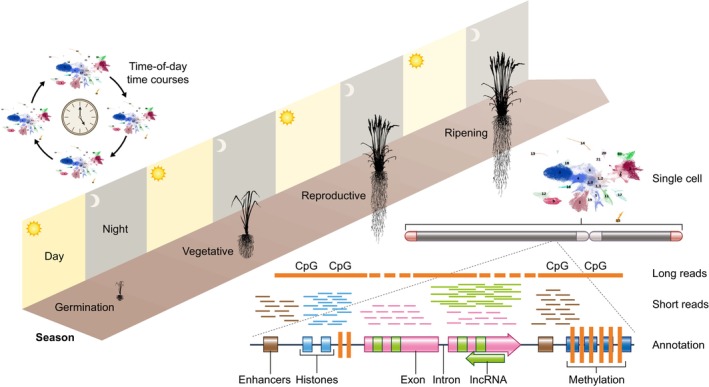
Time‐of‐day (TOD), spatial, and developmental ‘high‐definition genomics’ deliver both the breadth and the critical data types necessary for effective artificial intelligence (AI) modeling. The introduction of short‐read sequencing initially resulted in a wave of new low‐quality genomes, but quickly became the primary ‘microscope’ to look at the molecular level of the cell due to its counting nature. This resulted in an explosion of ‘Seq’ applications that enabled efforts such as the Encyclopedia of DNA Elements (ENCODE) project to define enhancers (brown), histone marks (blue), exons (pink), introns (black line), long noncoding RNA (lncRNA, green), and DNA methylation (CpG, orange). Now, many of these techniques have been modified for single‐cell applications (scRNAseq t‐sne (t‐distributed Stochastic Neighbor Embedding) pictured) providing the high‐resolution spatial information required for *in silico* modeling and ultimately the development of synthetic circuits and plants. In addition to single‐cell spatial information, which provides a static snapshot of the genome, it will be critical to collect temporal information such as time of day (sampling during both day (yellow) and night (gray)) and developmental (over the season; germination, vegetative, reproductive and ripening) to capture the dynamics of the system and response to an ever changing environment.

While the introduction of NGS led to a growing number of new genomes, it primarily enabled the development of molecular approaches to dissect previously untestable aspects of molecular biology (Koboldt *et al*., 2013). Since the initial innovation of NGS was to generate millions of short sequences instead of hundreds to thousands that were possible with Sanger sequencing, almost immediately it was recognized that these technologies could be used for counting applications such as RNA quantification (RNA‐seq), which exploded into a panoply of ‘Seq’ type molecular applications (‘Enseqlopedia’; Hadfield & Retief, 2018). The ability to probe almost all molecular aspects of a plant led to a dramatic increase in data rivaling that only found in model species, opening the opportunity to realize a plant's biology both spatially and temporally (Swift *et al*., 2021; Fig. 2).

Spatial genomics applications have changed the resolution of biological data almost overnight (Yin *et al*., 2023). Single‐cell sequencing makes it possible to probe both spatially and developmentally at the whole plant level (Lee *et al*., 2023; Denyer *et al*., 2024). However, the annotation of gene clusters in nonmodel species remains a challenge due to sparse cross‐species cell identity mapping, although approaches are being developed rapidly (Yan *et al*., 2022; Chau *et al*., 2024). Central to these efforts are tools for validating gene expression, ranging from adaptations of low‐throughput methods (Nobori *et al*., 2023b) to highly multiplexed spatial *in situ* hybridization technologies (Nobori *et al*., 2023a). These advancements are being unified through a collaborative initiative among scientists to build the Plant Cell Atlas, a comprehensive resource for plant cell identity and function (Rhee *et al*., 2019).

While spatial information increases our static molecular resolution, temporal information, both time of day (TOD) and developmental (over the season), are required to understand the dynamics of a plant (Oravec & Greenham, 2022). Green organisms from algae to higher plants partition their biology to coincide with the daily transitions of light and temperature, which enhances their ability to anticipate daily changes across seasons (Michael *et al*., 2003). Global whole‐plant transcriptomics has shown that nearly all genes in model plants such as *Arabidopsis* and rice are expressed in a TOD manner, aligning biological processes with seasonal environmental conditions (Michael *et al*., 2008; Michael, 2022), and these conserved processes across species provide a valuable reference for understanding networks that are critical targets for crop improvement (Filichkin *et al*., 2011; Ferrari *et al*., 2019). Since plants also respond to water and temperature stress in a TOD‐dependent manner, recent studies incorporating this temporal framework are reshaping our understanding of these essential stress response mechanisms (Greenham *et al*., 2017; Blair *et al*., 2019, 2022; Bonnot & Nagel, 2021; Tolsma *et al*., 2022). Integrating spatial, temporal and developmental data types at a single‐cell level will provide the resolution required to model a plant's response to its daily and seasonally changing environment (Fig. 2).

## IV. The *in silico* plant emerges

The increasing availability of molecular and phenotypic data, coupled with advances in computational power, has paved the way for the development of the first ‘*in silico* plant’. Lazebnik's call for a systematic and standardized language for molecular data was crucial not only for accurate modeling but also for cross‐species analyses beyond reference genomes, ultimately leading to the development of ontologies (Bard & Rhee, 2004). Controlled vocabularies have facilitated the integration of AI approaches, such as symbolic AI (e.g. pathway diagrams) and numeric AI (e.g. machine learning). These tools are now converging with LLMs, unlocking predictive capabilities across species (Agathokleous *et al*., 2024).

Machine learning has seen extensive applications across plant biology, ranging from genomic selection, which has revolutionized modern breeding (Meuwissen *et al*., 2001), to advancements in protein structure prediction (Jumper *et al*., 2021) and gene annotation (Stiehler *et al*., 2021). Initiatives such as Crops *in silico* (Marshall‐Colon *et al*., 2017) have brought together research groups to model complex networks, such as the circadian clock (Chew *et al*., 2022). These efforts represent substantial progress, but the next frontier in plant research lies in the application of LLMs, which aim to decode the molecular ‘language’ of biology and enhance our ability to design synthetic plants (Lam *et al*., 2024).

Advanced LLMs are already being used to predict critical genomic features such as polyadenylation sites, splice sites, open chromatin regions, and enhancers, which are key elements for synthetic plant design (Yang *et al*., 2024). Models such as DNABERT (Bidirectional Encoder Representations from Transformers) perform more robustly when trained on pangenomes and superpangenomes, improving their ability to generalize predictive tasks across diverse genomic features (Zhou *et al*., 2023). Furthermore, single‐cell Generative Pre‐trained Transformer (scGPT) demonstrates the potential to integrate symbolic and numeric AI, impute missing data, and elucidate regulatory networks (Cui *et al*., 2024). As these models evolve, their capacity to integrate vast datasets will transform plant system modeling, enabling tailored designs for synthetic plants and redefine crop improvement and synthetic biology.

## V. Designing the synthetic plant

**Fig. 3:**
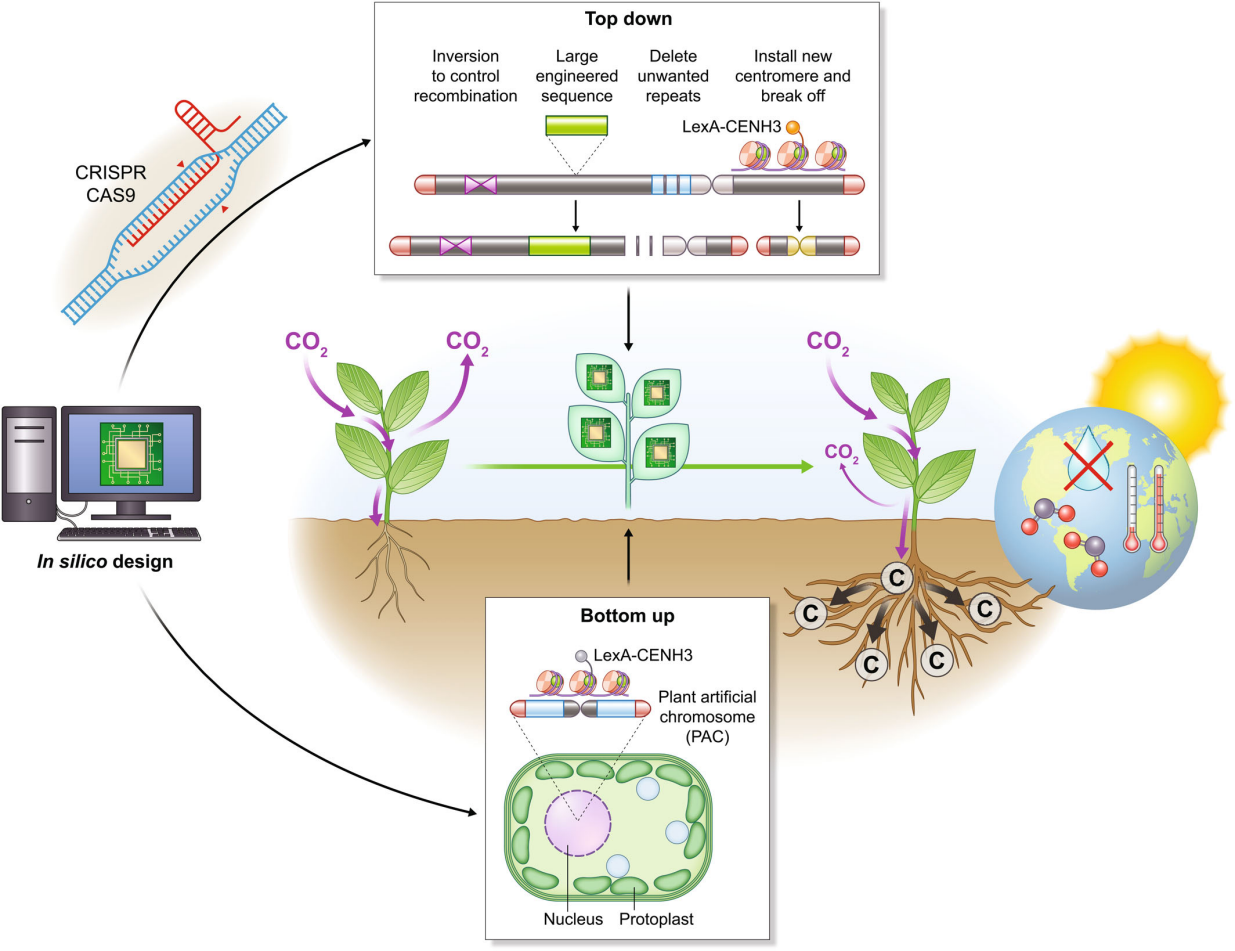
*In silico* models lay the foundation for designing and developing synthetic plants that can act as ‘environmental thermostats’ to help regulate the planet's climate. Amid a changing climate that impacts the availability of water (more and less), nutrients, and favorable growth temperature, there is a need to model plants *in silico* that will be able to thrive in these future conditions. Efforts such as crops *in silico* (https://cropsinsilico.org/) are paving the way for integrating molecular and phenotypic data across all scales (molecule, genome, photosynthesis/metabolite, organ, ecosystem). The advent of CRISPR (Clustered regularly interspaced short palindromic repeats) CAS9‐based editing has provided a robust method to ‘hack’ the genome that in combination with other editing tools enables the creation of inventions to control recombination, knock‐in of engineered genome regions, complete knock out (KO) of genes and repeat regions, and minichromosomes by installing centromeres or telomeres. These tools coupled to developments in our understanding of how centromeres are specified through *CENH3* (*HRT12*) and advances in cell biology have enabled the development of the first synthetic plants through ‘top‐down’ engineering by chromosome breaking and fusion. The future promises the opportunity to create synthetic plant chromosomes (PACs) through ‘bottom‐up’ engineering methods that will be installed into the nucleus through a protoplasting step. While technologies like PACs are not essential for realizing the first generation of synthetic plants, they could revolutionize plant engineering, enabling the creation of entirely new plant systems capable of performing functions we can only imagine today. Several ongoing efforts aim to leverage plants as ‘environmental thermostats,’ including RuBisCO optimization, C_3_‐to‐C_4_ pathway engineering, and CAM photosynthesis enhancement to improve water use efficiency (WUE). Additionally, novel approaches are being explored for food, fiber, and fuel engineering. One particularly promising target, highlighted in the figure, is altering root system architecture (RSA) to store captured carbon dioxide (CO_2_) as stable carbon forms in the soil for extended periods. Traditional breeding has predominantly focused on aboveground yield, leaving an opportunity to engineer plants with larger, deeper root systems capable of sequestering more carbon belowground. Roots naturally produce recalcitrant carbon compounds, such as suberin (cork), which have the potential to significantly extend the half‐life of carbon stored in soils. Plants with enhanced RSA that feature deeper, larger roots with higher levels of recalcitrant carbon could offer multiple benefits, including improving soil quality, reducing agricultural inputs, and increasing WUE. These innovations hold great promise for addressing climate challenges while enhancing agricultural sustainability.

Once we have the tools to build a model of a plant, and *in silico* designs for new genome features, we will need to ‘hack it’ (Kamoun, 2017; Fig. 3). The synthetic plant can be built in several ways: ‘bottom up’ by building new chromosomes, or ‘top down’ by editing or manipulating existing chromosomes (Puchta & Houben, 2024). Two distinct fronts are emerging to enable the generation of the first synthetic plants that mirror the thinking of Sophien Kamoun: the software needs to be manipulated, which means editing and circuit logic tools (de Lange *et al*., 2018; Brophy, 2022), as well as the hardware such as cells and chromosomes must be manipulated (Dawe, 2020).

Synthetic circuit design and implementation have been the most active area research in synthetic plant development (Vazquez‐Vilar *et al*., 2023; Ragland *et al*., 2024). Many circuit engineering studies were conducted in orthologous systems such as yeast (Havens *et al*., 2012), yet provided the momentum along with new editing technologies for *in planta* demonstrations (Khakhar *et al*., 2018). However, much effort has been placed on developing the toolkit for circuit design (Khan *et al*., 2022), such as synthetic promoters (Yang & Nemhauser, 2023). In a beautiful demonstration of circuit design and application to a specific plant phenotype, root system architecture (RSA) was modified *in planta* in a predictable fashion (Brophy *et al*., 2022).

Manipulating the hardware has proven more difficult since making large changes in chromosomes or installing chromosomes into nuclei present new challenges to the cell biology of plants (Puchta & Houben, 2024). The ‘holy grail’ of engineering is being able to control crossovers, and the first steps toward brute force changes have been accomplished leveraging editing techniques, which can be used for creating species barriers and potentially new species (Rönspies *et al*., 2022). In a model species that is particularly easy to manipulate such as moss, it has been shown that whole portions of the chromosome can be redesigned and installed as a first step to the first synthetic moss (SynMoss) (Chen *et al*., 2024). A top‐down approach to engineering was proposed by chromosome truncation (Yu *et al*., 2007), which was realized at an entirely new level recently with the installation of a *de novo* centromere that was specified epigenetically by CENH3 in maize (Dawe *et al*., 2023). If new centromeres can be installed, then the only bottle neck for bottom‐up engineering is physically installing a chromosome (or mini chromosome) into a plant cell.

## VI. Can a plant biologist fix a thermostat?

Plants have immense potential to capture and store carbon dioxide, serving as scalable and efficient ‘environmental thermostats’ to help regulate the planet's climate (Busch & Miller, 2022; Fig. 3). As the primary contributors to Earth's biomass (Bar‐On *et al*., 2018), plants have historically influenced climate change. For example, the freshwater fern *Azolla* is believed to have removed significant amounts of atmospheric carbon, contributing to a cooler Earth during the Eocene epoch (Brinkhuis *et al*., 2006). While significant efforts are underway to develop climate‐resilient plants with synthetic biology approaches (Archibald *et al*., 2023), it remains crucial to align scientific advancements with policy frameworks to ensure the sustainability of these initiatives (Gakpo *et al*., 2024). We are entering a new era where systems biology approaches can be effectively leveraged in conjunction with reductionist methods, creating a powerful synergy to tackle complex biological challenges (Williams & Auwerx, 2015). The next generation of plants must be developed with a comprehensive, forward‐thinking approach that integrates both perspectives to address the challenges posed by current and future climates (Yang *et al*., 2021).

## Competing interests

TPM is a cofounder of Cquesta, a company that works on crop root growth and carbon sequestration.

## Disclaimer

The New Phytologist Foundation remains neutral with regard to jurisdictional claims in maps and in any institutional affiliations.
